# Association between the Use of Backpack and Static Foot Posture in Schoolchildren with Static Pronated Foot Posture: A 36-Month Cohort Study

**DOI:** 10.3390/children8090800

**Published:** 2021-09-11

**Authors:** Pilar Alfageme-García, Julián Fernando Calderón-García, Alfonso Martínez-Nova, Sonia Hidalgo-Ruiz, Belinda Basilio-Fernández, Sergio Rico-Martín

**Affiliations:** 1 Nursing Departament, University Center of Plasencia, University of Extremadura, 10600 Plasencia, Spain; palfagemeg@unex.es (P.A.-G.); podoalf@unex.es (A.M.-N.); kirosony@unex.es (S.H.-R.); bbasfer@unex.es (B.B.-F.); 2 Nursing Department, Nursing and Occupational Therapy College, University of Extremadura, 10003 Cáceres, Spain; sergiorico@unex.es

**Keywords:** backpack, flat foot, foot index posture, neutral foot, pronated foot, schoolchildren

## Abstract

*Background:* Schoolchildren often spend a lot of time carrying a backpack with school equipment, which can be very heavy. The impact a backpack may have on the pronated feet of schoolchildren is unknown. *Aims:* The objective of this study was to evaluate the association of the backpack use on static foot posture in schoolchildren with a pronated foot posture over 36 months of follow-up. *Methods*: This observational longitudinal prospective study was based on a cohort of consecutive healthy schoolchildren with pronated feet from fifteen different schools in Plasencia (Spain). The following parameters were collected and measured in all children included in the study: sex, age, height, weight, body mass index, metatarsal formula, foot shape, type of shoes, and type of schoolbag (non-backpack and backpack). Static foot posture was determined by the mean of the foot posture index (FPI). The FPI was assessed again after 36 months. *Results:* A total of 112 participants used a backpack when going to school. Over the 36-month follow-up period, 76 schoolchildren who had a static pronated foot posture evolve a neutral foot posture. Univariate analysis showed that the schoolchildren using backpacks were at a greater risk of not developing neutral foot (odds ratio [OR]: 2.09; 95% CI: 1.08–4.09). The multivariate analysis provided similar results, where the schoolchildren using a backpack (adjusted OR [aOR]: 1.94; 95% CI: 1.02–3.82) had a significantly greater risk of not developing a neutral foot posture. *Conclusions:* A weak relationship was found between backpack use and schoolchildren aged from five to eleven years with static pronated feet not developing a neutral foot posture over a follow-up period of 36 months.

## 1. Introduction

In Spain, backpacks are widely used among schoolchildren. They often spend a lot of time carrying a backpack with school equipment that includes books, pencils, laptops, calculators, sports uniforms, water bottles, etc., which can be very heavy. Due to the loads being carried by schoolchildren, whose musculoskeletal systems are still maturing [1] and are undergoing rapid physical development [2], load carriage has been related to musculoskeletal injuries [3] and adult disability [4,5]. The angle of forward lean of the trunk is greater when carrying the load on the back than during normal walking [6], and the load is associated with back pain [7]. It has been shown that excessive loads increase the force and pressure under different foot areas [8], and they are the major factor influencing changes in gait patterns [9,10]. Both dynamic and static gait postural changes have been detected with external loads of more than twenty percent of the child’s corporal weight [11].

The feet play an important role in balance and movement in standing and walking [12,13]. Sex, age, genetics, shoes, body weight, and physical activity are some internal and external factors that affect the morphological and functional development of child’s foot [14,15,16].

Traditionally, the human foot is considered to be able to adopt three positions: neutral, supine, and prone positions. Foot pronation involves abduction (transverse plane), eversion (frontal plane), and dorsiflexion (sagittal plane), which are obtained by articulating the foot in different degrees of freedom. [17].

Flatfoot is a common and complex foot malformation seen in children of both sexes, and it often worries parents [18]. A standard definition of flatfoot does not exist; nevertheless, it is characterized by a pronated foot that shows foot abduction at the talonavicular joint, the collapse of the medial longitudinal arch, and hindfoot valgus (subtalar joint eversion) [19,20]. Frequently, some schoolchildren have physiologic flatfeet, which is mostly flexible and asymptomatic [21]. Sometimes, flatfoot can cause comorbidities [22], affecting the quality of life [23]. Moreover, these disorders can be related to foot problems, including foot pain [24,25], hammertoes [26], hallux abducto valgus [27], and other lower extremity injuries [28]. On the other hand, hypermobility of the first ray can be associated as a primary cause of hallux valgus and different types of metatarsalgia [29].

There are discrepancies in the treatment of asymptomatic pediatric flatfoot [30], as there is no scientific evidence to justify that orthoses improve symptoms or correct flatfoot deformities [21,31]. Currently, in children with flexible and asymptomatic flatfoot, treatment frequently requires education for the parents and supervision of foot development.

In a previous study, the relationship of backpack use on static foot posture in schoolchildren with a neutral foot posture was investigated [32]. Over a follow-up period of 3 years, a positive association was observed between the risks of developing static pronated foot with backpack use in schoolchildren aged from five to eleven years with neutral foot posture. However, no previous research studied the association between static pronated foot posture and backpack in schoolchildren. The objective of this study is to evaluate the association of the backpack use on static foot posture in schoolchildren with a pronated foot posture over 36 months of follow-up.

## 2. Materials and Methods

### 2.1. Study Design, Setting, and Participants

The method employed in this observational longitudinal and prospective study was similar to that used in a previous study carried out by our research group [32] according to the STROBE guidelines for reporting observational studies [33]. Therefore, this paper is a continuation of the previously published study [32] and was designed accordingly to the Declaration of Helsinki. The ethics committee of the University of Extremadura approved the study protocol (ID: 59/2012; Approval date: 26 May 2015). Prior to the start of the study, parents were informed about the details of the research and subsequently signed an informed consent form to allow their children to participate.

Schoolchildren were recruited from fifteen different schools in Plasencia (Spain). March 2014 was the start date of recruitment. The inclusion criteria for our study are similar to the previously published study [32], except that the schoolchildren had to have an FPI score indicating static pronated foot.

### 2.2. Study Variables

The following parameters were collected and measured in all children included in the study: sex, age, height, weight, body mass index, metatarsal formula, foot shape, type of shoes, sedentary lifestyle, and type of schoolbag (non-backpack and backpack). Non-backpack included includes the use of a briefcase or rolling-style backpacks. Schoolchildren were categorized into four categories based on their age-adjusted BMI (underweight, normal, overweight, and obese) according to the classification system published by Orbegozo [34]. Schoolchildren who did not engage in physical activity outside of school were identified as sedentary. The assessment of the foot shape (Egyptian, Greek, and Square) and metatarsal formula (Index plus, Index minus, and Index plus minus) were performed by an experienced podiatrist according to the criteria established by Young C et al. [35].

**Figure 1:**
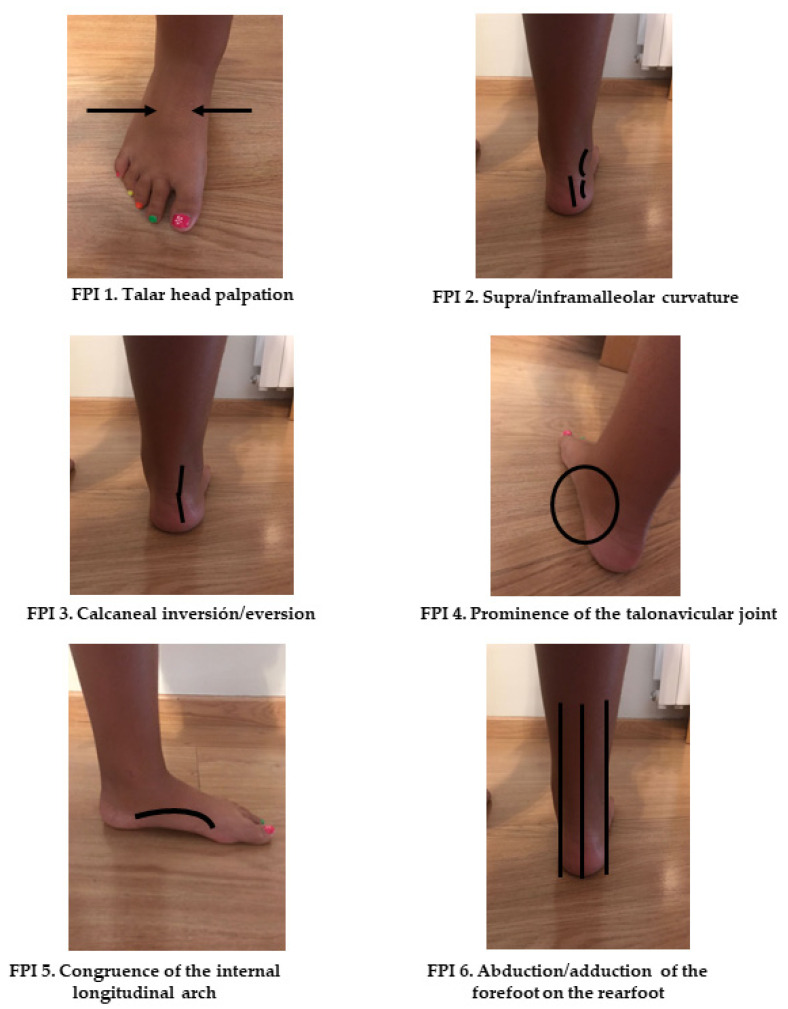
The six criteria of Foot Posture Index.

FPI measurements were assessed at baseline and repeated 36 months later, following the methodology of previous studies [32,36,37,38,39] by the same podiatrist (P.A.G). The FPI results shown in the study are for the right foot, which was chosen by randomization, although data from both feet were evaluated [32]. This index assesses the following criteria (Figure 1): talar head palpation, supra/inframalleolar curvature, calcaneal frontal plane position, prominence in the region of the talonavicular joint, congruence of the medial longitudinal arch, and abduction/adduction of the forefoot on the rearfoot. For each criterion, the score ranges from −2 to +2. The total score obtained from the sum of the six criteria classifies the static posture of the foot into: highly pronated (10 to 12), pronated (6 to 9), neutral (0 to 5), supinated (−4 to −1), and highly supinated (−12 to −5).

### 2.3. Statistical Methods

IBM SPSS v.24 (IBM Corporation, Armonk, NY, USA) software was used to perform the statistical analysis (performed by J.F.C.-G. and S.R.-M.). Categorical variables are reported as frequencies (%), and the continuous variables are as averages ± standard deviations. Categorical variables between the backpack and non-backpack group were compared using the chi-square test or Fisher’s exact test (if the frequency observed in any of the groups was less than 5). Normal distribution was considered when *p* > 0.05 was obtained in the Kolmogorov–Smirnov test. The continuous variables were compared by Student’s t-test for normal distribution (paired samples: FPI score baseline vs. FPI score 36 months later; independent samples: backpack vs. non-backpack) or Wilcoxon test (paired samples) and Mann–Whitney U test (independent samples) by non-normal distribution. On the other hand, uni and multivariate logistic regression analysis were performed to evaluate the relationship between independent variables and dependent variable (the change from pronated foot to neutral foot within the 36 months of follow-up). The odds ratios (ORs) and their corresponding 95% confidence intervals (CI) were calculated when *p* < 0.10 in the univariate analysis. Independent variables with *p* < 0.10 in univariate analysis were included in the multivariate analysis.

## 3. Results

**Figure 2:**
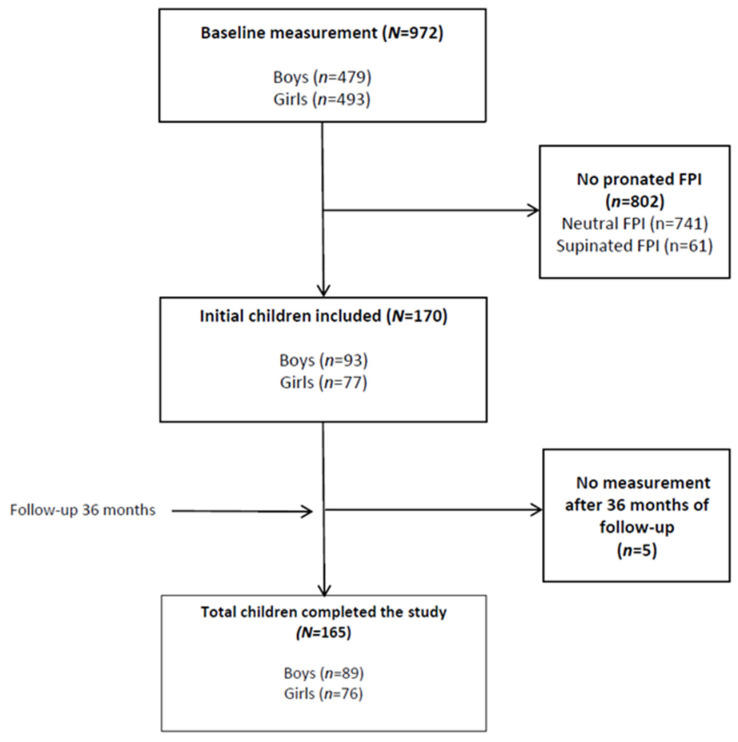
Schoolchildren selection process.

A total of 165 schoolchildren were included (76 girls and 89 boys). Of these schoolchildren, 112 (67.8%) used a backpack to go to school. Figure 2 illustrates the schoolchildren selection process. A total of 802 of the initial schoolchildren did not present FPI scores that indicated static pronated foot. Five schoolchildren were excluded because they were lost to follow-up and/or had any exclusion criteria (pain during exploration or lower-limb injury occurring within the 36 months of follow-up).

The mean age of the schoolchildren was 8.37 ± 1.56 years (range from five to eleven years), and the mean BMI was 19.01 ± 3.46 kg/m2. Table 1 shows the participant’s main characteristics according to the use of backpacks. Significant differences were found between the non-backpack and backpack subjects aged < 7 years (*p* = 0.015), those aged > 9 years (*p* = 0.025), those who used sport shoes (*p* = 0.016), and those who used ballet flats (*p* = 0.008). No significant differences were observed for the rest of the analyzed variables.

The average FPI score at baseline was 2.87 ± 1.15 (range from 6 to 12). The prospective study findings for the each of six criteria and total FPI scores are shown in Table 2. In all schoolchildren, after 36 months, the scores for each FPI criteria (*p* < 0.05) and the total FPI score (*p* < 0.001) significantly decreased. In the backpack group, there was a significant decrease in the total FPI score (*p* < 0.001) and in the following four FPI criteria: curves at the malleolus (*p* = 0.007), inversion/eversion of the calcaneus (*p* = 0.005), congruence of the medial arch (*p* = 0.002), and abd/adduction of the forefoot on the rearfoot (*p* < 0.001). On the other hand, in the non-backpack participants, after 36 months, the total FPI score (*p* < 0.001) and the scores for two (of six) FPI criteria, TNJ prominence (*p* = 0.046), and ab/adduction of the forefoot on the rearfoot, significantly reduced (*p* = 0.003).

Over the 36-months follow-up period, 76 (46.1%) schoolchildren who had a pronated foot posture developed a neutral foot posture. Table 3 shows predictor variables studied for the change from a static pronated to a static neutral foot posture. Based on the univariate analysis, the schoolchildren using backpacks had an increased risk of not developing a static neutral foot posture (odds ratio [OR]: 2.09 95% CI: 1.08–4.09). Similar results were provided in the multivariate analysis, where the schoolchildren using a backpack (adjusted OR [aOR]: 1.94; 95% CI: 1.02–3.82) had an increased risk of not developing a static neutral foot posture. The rest of the variables included in the analysis were not identified to be predictors of individuals with a pronated foot posture not developing a neutral foot posture.

## 4. Discussion

In this observational and longitudinal study, the relationship between the changes in static foot posture and backpack use in schoolchildren with static pronated foot posture over a 36-months follow-up were studied. Our findings suggest that backpack use might be related to a pronated foot posture being maintained and to the schoolchildren with a static pronated foot posture not developing a static neutral foot posture.

Pronation is a complex joint movement that occurs in the transverse, frontal, and sagittal planes [40]. It involves abduction (transverse plane), dorsiflexion (sagittal plane), and eversion (frontal plane), which are obtained by articulating the foot in different degrees of freedom. In cases of pronated foot, the peak pressure is also found in the central region of the forefoot, where the excessive pronation generates hypermobility of the first radius displacing the loads increasing the plantar pressure on the head of the second metatarsal [41,42,43,44]. First ray hypermobility, if present, could be able to affect our results. In children practicing ballet, this hypermobility has been associated with hereditary anatomical factors and incorrect execution of the technique [45]. The main flatfoot’s characteristics are hindfoot valgus (subtalar joint eversion), collapse of the medial longitudinal arch, and foot abduction at the talonavicular joint manifested by a pronated foot.

Backpack use is widespread among Spanish schoolchildren. In the present investigation, over 67% of participants carried a backpack and the load was probably greater than the recommended limits, so this situation could result in a future public health problem [44]. Both dynamic and static gait postural changes have been detected with external loads of more than twenty percent of the child’s corporal weight [11]. Excessive loads increase the force and pressure under different foot areas [8]. The total load carried is the major factor influencing changes in gait patterns [9,10,46]. Carrying the backpack raises the maximum plantar pressures during upright stance, which are already elevated in this population, and alters normal plantar pressure patterns, particularly in the forefoot region in static stance [46]. These results have been similarly found in many non-obese and obese schoolchildren [47]. In university students, it has been found that the trunk position and the muscle activity depend on the backpack weight [48]. This study reported that the rectus abdominal muscular activity rose significantly when loads exceeded ten percent of corporal weight.

The effects of backpack use on foot function and structure have previously been studied in soldiers [49]. The major findings of this research showed the relationship of backpack use with a decrease in stride length and an increase in cadence. In addition, the use of backpacks was associated with increased ankle and hip ranges of movement, amplified trunk flexion angle, and augmented ground reaction forces. In our study, binary logistic regression indicated that BMI, sex, age, sedentariness, foot shape, metatarsal formula, and shoe type did not influence the change from a pronated to neutral foot posture in the subjects analyzed. On the other hand, the use of the backpack did have an influential effect on the non-development of neutral foot posture, according to multi and univariate analyses. This cohort study is the first to evaluate the changes that can happen in children aged from five to eleven years with a static pronated foot, so it cannot be compared with other studies. Our finding indicated that there was a weak association between the use of a backpack and children with a pronated foot posture not developing a static neutral foot posture over a follow-up period of 36 months. This association can be due to the fact that carrying the backpack while gaiting is associated with a decrease in stride length and an increase in cadence, increased ankle and hip ranges of movement, amplified trunk flexion angle, and augmented ground reaction forces [49]. Previously, our research group investigated the backpack effects on foot posture in 627 schoolchildren with a static neutral foot posture [32]. A positive association was observed between the risks of developing static pronated foot with backpack use in schoolchildren aged from 5 to 11 years with static neutral foot posture. However, according to the risk of developing supinated foot, this relationship was found.

In the pediatric population, there is a predominance of pronated and neutral foot postures; in addition, sex influences the FPI due to ligament and muscle differences [50,51]. In our study, sex did not influence the results. Mickle KJ et al. [14] reported that structural changes in foot anatomy in overweight/obese children might cause adverse effects throughout childhood and into adulthood.

Contrary to our study, the findings of observational studies have found a relationship between pronated foot and body mass index; however, the analyses were performed using a single plane and different methods of measurement, such as the footprint [15,52,53,54,55]. We measured the Foot Posture Index, which shows the posture of the foot in the sagittal, frontal, and transverse planes and includes all functional units of the foot (forefoot, midfoot, and rearfoot).

The main limitations of our study were the following: (1) the study design was observational, and thus no causal relationships can be reported; (2) this paper provides measurements taken only at baseline and after 36 months. Probably, measurements every 6–12 months would have yielded more information, especially according to the changes in the static posture of the pediatric foot during the foot’s development; (3) all children were recruited from a single city (Plasencia); therefore, the results may not be comparable to children of other areas; (4) the backpack weight was not assessed because the study was not originally designed to evaluate the effect of backpack use on static foot posture change. This limitation may have influenced the study results. Finally, we also did not consider the duration of backpack use and its position on the backs of the children studied.

## 5. Conclusions

In our study, over a follow-up period of 36 months, backpack use was weakly associated with the non-development of a static neutral foot posture in those children who previously had a pronated foot posture in statics.

## Figures and Tables

**Table 1:** Characteristics of the schoolchildren stratified by backpack use.

	All Participants (*n* = 165)	Backpack Participants (*n* = 112)	Non-Backpack Participants (*n* = 53)	*p*-Value
Age (years)	8.37 ± 1.56	8.61 ± 1.52	7.86 ± 1.53	0.004
Age categorized, years (%)				
<7 years	37 (22.4%)	19 (17.0%)	18 (34.0%)	0.015
7–9 years	64 (38.8%)	43 (38.9%)	21 (39.6%)	0.880
>9 years	64 (38.8%)	50 (44.6%)	14 (26.4%)	0.025
Gender male (%)	89 (53.9%)	60 (53.6%)	29 (54.7%)	0.890
BMI (kg/m^2^)	19.01 ± 3.46	19.11 ± 3.29	18.79 ± 3.8	0.578
Overweight–obesity (%)	11 (6.7%)	8 (7.1%)	3 (5.7%)	0.999
Sedentary (%)	73 (44.2%)	49 (43.8%)	24 (45.3%)	0.853
Metatarsal formula (%)				
Index Plus	37 (22.4%)	28 (25.0%)	9 (17.0%)	0.249
Index Plus Minus	96 (58.2%)	60 (53.6%)	36 (67.9%)	0.081
Index Minus	32 (19.4%)	24 (21.4%)	8 (15.1%)	0.337
Foot shape (%)				
Greek foot	55 (33.3%)	38 (33.9%)	17 (32.1%)	0.814
Square foot	84 (50.9%)	57 (50.9%)	27 (50.9%)	0.995
Egyptian foot	26 (15.8%)	17 (15.2%)	9 (17.0%)	0.767
Shoe type				
Sport	117 (70.9%)	86 (76.8%)	31 (58.5%)	0.016
Moccasin	13 (7.9%)	7 (6.3%)	6 (11.3%)	0.259
Ballet flats	28 (17.0%)	13 (11.6%)	15 (28.3%)	0.008
Mary Janes	7 (4.2%)	6 (5.4%)	1 (1.9%)	0.302

Data are reported as average ± standard deviation and frequencies (%). Abbreviations: BMI—body mass index.

**Table 2:** Total score for FPI and those for the six criteria stratified by backpack use.

	All Participants			Backpack Participants			Non-Backpack Participants		
	Baseline	36 Months	*p*-Value	Baseline	36 Months	*p*-Value	Baseline	36 Months	*p*-Value
Talar head palpation	1.27 ± 0.51	1.19 ± 0.52	0.034	1.27 ± 0.50	1.20 ± 0.51	0.158	1.28 ± 0.57	1.17 ± 0.54	0.083
Curves at malleolus	1.10 ± 0.45	0.94 ± 0.58	0.003	1.10 ± 0.46	0.92 ± 0.60	0.007	1.11 ± 0.42	0.98 ± 0.53	0.180
Inversion/Eversion calcaneus	1.07 ± 0.48	0.89 ± 0.48	0.001	1.07 ± 0.47	0.89 ± 0.49	0.005	1.06 ± 0.49	0.89 ± 0.46	0.060
TNJ prominence	1.10 ± 0.50	0.94 ± 0.57	0.006	1.06 ± 0.45	0.95 ± 0.51	0.058	1.17 ± 0.61	0.92 ± 0.67	0.046
Congruence of medial arch	1.08 ± 0.46	0.92 ± 0.51	0.002	1.11 ± 0.45	0.94 ± 0.48	0.003	1.02 ± 0.50	0.89 ± 0.54	0.196
Ab/adduction forefoot–rearfoot	1.27 ± 0.55	0.98 ± 0.49	<0.001	1.29 ± 0.54	0.99 ± 0.51	<0.001	1.21 ± 0.56	0.94 ± 0.45	0.003
Total SCORE FPI	6.87 ± 1.15	5.86 ± 1.79	<0.001	6.88 ± 1.18	5.89 ± 1.79	<0.001	6.85 ± 1.11	5.79 ± 1.82	<0.001

Data are reported as average ± standard deviation. Abbreviations: Ab—abduction; FPI—foot posture index; TNJ—talonavicular joint.

**Table 3:** Predictors of individuals with pronated foot not developing a neutral foot posture.

			Univariate Analysis		Multivariate Analysis	
	FPI Neutral *n* = 76	FPI Pronated *n* = 89	OR (CI%95)	*p*-Value	aOR (CI%95)	*p*-Value
Backpack	45 (59.2%)	67 (75.3%)	2.09 (1.08–4.09)	0.028	1.94 (1.02–3.82)	0.048
Age (year)	-	-	0.95 (0.78–1.16)	0.629	-	-
Age categorized						
Age < 7 years	16 (21.1%)	21 (23.6%)	1.15 (0.55–2.42)	0.696	-	-
Age 7–9 years	28 (36.8%)	36 (40.4%)	1.16 (0.62–2.18)	0.635	-	-
Age > 9 years	32 (42.1%)	32 (36.0%)	0.77 (0.41–1.44)	0.419	-	-
Gender male (%)	40 (52.6%)	49 (55.1%)	1.10 (0.59–2.03)	0.755	-	-
BMI (kg/m^2^)	-	-		0.470		
Overweight–obesity (%)	4 (5.3%)	7 (7.9%)	1.53 (0.43–5.46)	0.550	-	
Sedentary (%)	31 (40.8%)	42 (47.2%)	1.29 (0.69–2.40)	0.409	-	-
Metatarsal formula (%)						
Index Plus	14 (18.4%)	23 (25.8%)	1.54 (0.72–3.26)	0.255	-	-
Index Plus Minus	46 (60.5%)	50 (56.2%)	0.83 (0.44–1.55)	0.573	-	-
Index Minus	16 (21.1%)	16 (18.0%)	0.82 (0.38–1.78)	0.619		
Foot shape (%)						
Greek foot	23 (30.3%)	32 (36.0%)	1.29 (0.67–2.48)	0.439	-	-
Square foot	44 (57.9%)	40 (44.9%)	0.59 (0.32–1.10)	0.097	0.57 (0.30–1.08)	0.090
Egyptian foot	9 (11.8%)	17 (19.1%)	1.75 (0.73–4.21)	0.202	-	-
Shoe type						
Sport	51 (67.1%)	66 (74.2%)	1.40 (0.71–2.76)	0.320	-	-
Moccasin	7 (9.2%)	6 (6.7%)	0.71 (0.22–2.22)	0.557	-	-
Ballet flats	17 (22.4%)	11 (12.4%)	0.48 (0.21–1.12)	0.088	0.56 (0.23–1.33)	0.190
Mary Janes	1 (1.3%)	6 (6.7%)	5.42 (0.63–46.07)	0.125	-	-

Data are reported as frequencies (%) and OR (95% CI). Abbreviations: aOR—adjusted odds ratio; BMI—body mass index; CI—confidence interval; FPI—foot posture index; OR—odds ratio.
